# Medication Overuse Headache Successfully Treated by Japanese Herbal Kampo Medicine, Yokukansan

**DOI:** 10.7759/cureus.18326

**Published:** 2021-09-27

**Authors:** Masahito Katsuki, Shin Kawamura, Kenta Kashiwagi, Akihito Koh

**Affiliations:** 1 Department of Neurosurgery, Itoigawa General Hospital, Itoigawa, JPN; 2 Department of Neurology, Itoigawa General Hospital, Itoigawa, JPN

**Keywords:** japanese herbal medicine, kampo medicine, chronic migraine, chronic headache, alternative medical therapies, elderly, medication overuse headache (moh), yokukansan (tj-54)

## Abstract

Medication overuse headache (MOH) usually resolves after the overuse is stopped. However, it can be challenging to prescribe common prophylactic medications when patients are old or have concerns about the side effects of Western prophylactic medications. As an alternative therapy, traditional Japanese herbal Kampo medicine can be used. One of them, yokukansan (TJ-54), is often used for behavioral and psychological symptoms of dementia in Japan. Recently, it has been reported as an alternative medication for episodic, chronic, or MOH. We herein report a MOH in an older man already taking antihypertensive drugs. His MOH was successfully relieved by TJ-54 instead of the common prophylactic medications. His headache and nausea were relieved on day four of the treatment. After that, he did not need any analgesic drugs. Of course, we should pay attention to the side effects, pseudoaldosteronism, but TJ-54 may be one of the alternative treatment therapies for MOH.

## Introduction

Medication overuse headache (MOH), coded 8.2 according to the International Classification of Headache Disorders 3rd edition (ICHD-3) [1], is a condition in which headaches occur 15 or more days per month in a patient with a pre-existing primary headache and develops as a consequence of regular overuse of acute or symptomatic headache medication (on 10 or more or 15 or more days per month, depending on the medication) for more than three months. The annual prevalence of MOH is 1-2% and it is common in middle-aged people and women. The headache usually resolves after the overuse is stopped [2]. According to the Japanese Clinical Practice Guideline for Chronic Headache 2013 [3], the treatment strategy is as follows: (a) discontinue the overused medication, (b) treat the headache after discontinuing the overused medication, and (c) administer prophylactic medications.

However, it can be challenging to prescribe common prophylactic medications like anticonvulsants and antidepressants for older MOH patients because of the side effects from prophylactic medications, such as drowsiness and allergies. As an alternative therapy, traditional Japanese herbal Kampo medicine can be used [3,4]. Yokukansan (TJ-54) is a traditional Japanese herbal Kampo medicine used to treat neurosis, insomnia, night crying, and irritability, and/or agitation in infants and is approved for prescription in Japan [5]. It is also often used in Japan for behavioral and psychological symptoms of dementia [6]. Recently, TJ-54 has been reported as an alternative therapy for episodic headache [7], chronic headache [5], or MOH [8,9], but such reports remain few. We herein report a MOH in an older man already taking antihypertensive drugs. His MOH was successfully relieved by TJ-54 instead of the common prophylactic medications.

## Case presentation

**Figure 1:**
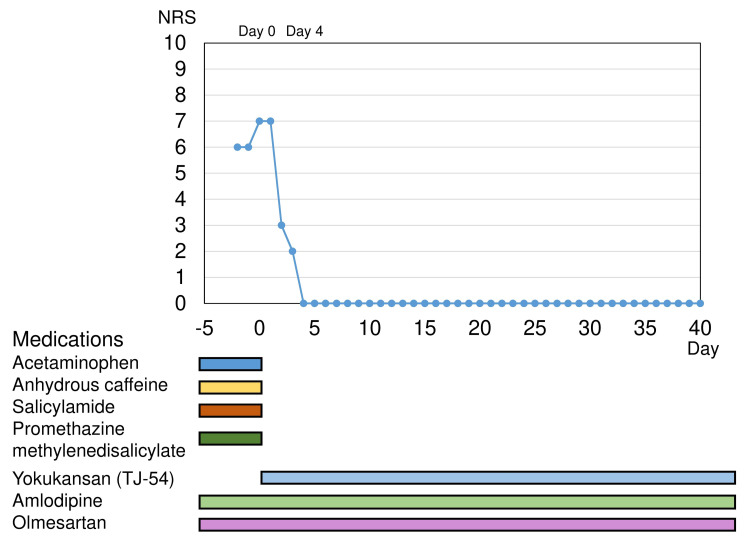
Headache severity and the medication intake After stopping the combination-analgesic medication and starting yokukansan (TJ-54), the severity gradually improved. NRS: numerical rating scale

An 85-year-old man presented a chronic headache with a numeric rating scale (NRS) of 6/10 to our neurosurgical outpatient ward. The headache was not pulsatile but unilateral. There was no headache aggravation or avoidance of physical activity due to the headache, but nausea was present. The attacks lasted throughout the daytime. The patient had suffered from migraines since the age of 50 years, and he had been using over-the-counter medications. For the past two years, he had been taking three times daily 550-mg acetaminophen, 60-mg anhydrous caffeine, 270-mg salicylamide, and 13.5-mg promethazine methylene disalicylate as combination-analgesic medication from his family doctor. He also had hypertension and insomnia, so he took 20-mg olmesartan, 2.5-mg amlodipine, and 0.25-mg brotizolam as needed. Other than antihypertensive medication, he had never tried any prophylactic medications. The Hasegawa dementia scale-revised score was 26/30. The head CT and laboratory tests revealed that the headache did not seem to be secondary, so we diagnosed non-steroidal acetaminophen-overuse headache (ICHD-3 code 8.2.3.1), combination-analgesic-overuse headache (ICHD-3 code 8.2.5), and chronic migraine (ICHD-3 code 1.3). We stopped all the analgesic medications and let him keep a headache diary. Considering his older age and the side effects of using anticonvulsants or antidepressants as common prophylactic medications, we prescribed three packets of TJ-54 per day. His headache and nausea were relieved on day four of the treatment. He did not need any anti-inflammatory drugs in these 40 days, and we will continue TJ-54 (Figure 1). We are considering tapering off the dosage of TJ-54 every two weeks, ending the course in about two months.

## Discussion

We presented the case of an elderly man with MOH who was successfully treated using TJ-54. We used TJ-54 instead of common prophylactic medication because of the age of the patient and concerns regarding side effects. Although this is a single case report, TJ-54 seemed effective for MOH. Here, we review Kampo medicine for MOH and discuss how TJ-54 acts and what we should pay attention to.

TJ-54 for MOH

TJ-54 contains *Atractylodes lancea* rhizome (4.0  g), *Poria cocos* sclerotium (4.0  g), Cnidium rhizome (3.0  g), Uncaria hook (3.0  g), Japanese angelica root (3.0  g), Bupleurum root (2.0  g), and Glycyrrhiza (kanzo) (1.5  g). It is not commonly used to treat headaches and has not yet been recommended in the Japanese Clinical Practice Guideline for Chronic Headache 2013 [3]. However, in Japan, we often use TJ-54 to relieve the symptom related to dementia [6], and many Japanese clinicians may be familiar with its prescription. Previously, Mitsufuji reported three cases of MOH that were successfully treated by TJ-54 [8,9]. Of the three cases, one distrusted Western medication, one had irritability, and one had anxiety and depression. The first case continued TJ-54 for 16 days. The second and third cases continued TJ-54 but they did not clearly describe when TJ-54 would be stopped.

The mechanism of TJ-54 for headaches remains unclear. However, the following mechanisms have been reported: (a) suppression of neuronal excitation in the glutamatergic system through suppression of glutamate release action, which affects N-methyl-D-aspartic acid receptors, activation of glutamate transporters, adjustment of glutamate uptake, and suppression of increases in the extracellular glutamate level; (b) increased extracellular serotonin level in the serotonergic central nervous system through partial agonist effect on 5-HT1A receptors and downregulation of 5-HT1A receptors; (c) inhibition of orexin-A secretion; (d) anti-inflammatory effects; and (e) suppression of the microglia’s activation in the spinal tract in the trigeminal nerve, which is important for the development of chronic migraine from episodic migraine [5,8,9]. These mechanisms collaboratively affect some of the central mechanisms in the trigeminovascular theory of migraine, and TJ-54 may relieve pain. Further research is needed.

Factors related to TJ-54 effectiveness

Kimura investigated the characteristics of episodic headaches that are relieved by TJ-54. Factors such as a decrease of painful eye sensation, back stiffness, eyestrain, and irritability were significant indicators of headache improvement. The three symptoms were the best subset of explanatory variables [7]. Our case had MOH, not an episodic headache, so further investigation is needed on what MOH patients are responsive to in TJ-54.

Side effects of TJ-54

Pseudoaldosteronism caused by Glycyrrhiza in TJ-54 is something to be aware of. According to the Japanese Adverse Drug Report (JADER) Database, the reporting odds ratio was 2.4 compared to other Glycyrrhiza-containing Kampo medicines. The dose amount, older age, low body weight, dementia are risk factors for pseudoaldosteronism [10]. Therefore, the TJ-54 dosage should be tapered according to symptoms, and a careful follow-up with a blood test will be needed. Also, the optimal duration of TJ-54 treatment for MOH should be investigated.

Other Kampo medicines for MOH

According to the Japanese Clinical Practice Guideline for Chronic Headache 2013, goshuyuto (TJ-31), keishininjinto (TJ-82), chotosan (TJ-47), kakkonto (TJ-1), and goreisan (TJ-17) are mentioned as empirically effective medications for headache treatment. We previously reported the efficacy of combined Kampo medicine treatment for a woman with MOH. The patient’s migraine was associated with weather, menopause, and muscle tension, so we prescribed goreisan (TJ-17), goshuyuto (TJ-31), and kakkonto (TJ-1) for each characteristic of the headache [11]. Goreisan (TJ-17) may activate the glymphatic flow correctly and promote the drainage of inflammatory substances by regulating the aquaporin 4 channel, thereby suppressing the headache [12,13]. Goshuyuto (TJ-31) may adjust serotonin levels and vessel constriction appropriately [14,15], and raising serotonin levels beforehand by goshuyuto [16] may suppress the hyper-reactivity of serotonin receptors for serotonin, leading to the prevention of migraine attack. Kakkonto (TJ-1) may relieve tension-type headaches by upregulating blood flow in the shoulder muscles [17].

Akaishi used tokishakuyakusan (TJ-23) for a 42-year-old MOH woman and successfully relieved her headache [18]. Tokishakuyakusan is hypothesized to increase the estrogen and progesterone secretion from the ovary, possibly by activating the hypothalamic‐pituitary‐gonadal axis. In addition, daisaikoto (TJ-8), shosaikoto (TJ-9) [19], tokishigyakukagosyuyushokyoto (TJ-38) [20] were also reported as useful alternative therapies for MOH as case reports. Further study on Kampo medicine for MOH is needed.

Our case report may lead to further robust studies like a randomized control trial using TJ-54 for MOH, which can help us have more options in treating patients like this. However, we need more data regarding traditional Japanese Kampo medicines. If these medications are found effective in further studies, we would also like to use them in treatment outside Japan.

## Conclusions

We presented the case of MOH in an older man who was successfully treated using TJ-54, considering the old age and the side effect of other Western prophylactic medicines. Although this is a single case report, TJ-54 seemed effective for MOH treatment. Of course, we should pay attention to the side effects, pseudoaldosteronism, but TJ-54 could be considered as an alternative treatment therapy for MOH.
